# Size-Fractionated Net Primary Production Distribution and Its Environmental Control in the East China Sea During Winter

**DOI:** 10.3390/biology15120905

**Published:** 2026-06-09

**Authors:** Jiahong Cheng, Chenggang Liu, Yuming Cai, Hongchang Zhai, Wei Zhang, Minhui Su, Qiang Hao

**Affiliations:** 1 Second Institute of Oceanography, Ministry of Natural Resources, Hangzhou 310012, China; chengjh@sio.org.cn (J.C.); liu_cg@vip.163.com (C.L.); andrewcai73@126.com (Y.C.); pianhe@163.com (H.Z.); zhangwei2020@sio.org.cn (W.Z.); minhuisu@zju.edu.cn (M.S.); 2 Key Laboratory of Marine Ecosystem Dynamics, Ministry of Natural Resources, Hangzhou 310012, China; 3 Key Laboratory of Nearshore Engineering Environment and Ecological Security of Zhejiang Province, Second Institute of Oceanography, Ministry of Natural Resources, Zhoushan 310012, China; 4 State Key Laboratory of Submarine Geoscience, Hangzhou 310012, China

**Keywords:** size-fractionated primary production, light exposure, P^B^_opt_, light limitation, winter East China Sea, satellite-based primary production model

## Abstract

Phytoplankton primary production (PP) underpins marine ecosystems. In winter marginal seas, the magnitude and size structure of PP not only sustain overwintering zooplankton but also shape larval fish survival and fishery resources in the following year. We conducted two cruises in the fish overwintering grounds of the East China Sea shelf to investigate the spatial distribution, size structure, and environmental controls of net primary production (NPP). Winter NPP was generally low relative to the annual range. Nutrient concentrations at most stations exceeded potential limitation thresholds, whereas the mixed-layer mean light exposure (LE) fell below the light-saturation threshold at most stations, indicating that insufficient light availability was primarily associated with sub-saturating light conditions of low winter productivity. Among size classes, the nano-sized fraction dominated NPP, followed by the pico-sized fraction, while the micro-sized fraction contributed least; however, the relative contribution of the micro-sized fraction increased in February. Measured values of two key parameters widely used in satellite-based NPP models—PBopt (optimal chlorophyll-specific carbon fixation rate) and F (a dimensionless light-related factor for the vertical distribution of primary production)—were both lower than model predictions, and the magnitude of deviation varied with water depth and mixing conditions. These findings refine our understanding of biogeochemical processes in overwintering grounds of winter marginal seas.

## Simple Summary

Microscopic algae in the ocean produce oxygen and form the base of the marine food web through a process called primary production, in which they use sunlight to convert carbon dioxide into organic matter. Understanding winter primary production is particularly important in the East China Sea, which serves as a critical overwintering and spawning ground for several commercially important fish species. This study measured primary production during two winter research cruises and examined how different sizes of algae contribute to total production. We found that winter production was low, primarily because strong wind-driven mixing of the water column reduced the amount of light available to algae. Among the three size groups of algae studied, medium-sized algae dominated production, while the contribution of the largest algae increased from early to late winter. This seasonal shift toward larger phytoplankton implies a potential alteration in the trophic structure of the pelagic food web. If such a shift leads to more efficient energy transfer through the food chain, it could, in principle, influence the prey field available to higher trophic levels, including overwintering fish populations. However, this hypothesis remains to be tested with concurrent measurements of zooplankton and fish larvae. We also found that widely used satellite models overestimated the photosynthetic capacity of winter algae. These findings help improve our understanding of winter ocean productivity and can contribute to better fishery management and more accurate satellite-based monitoring of ocean health.

## 1. Introduction

Primary production converts inorganic carbon into organic carbon in the ocean, determining ecosystem energy input and carbon sequestration potential [1,2]. Total primary production alone, however, cannot adequately capture the complexity of marine carbon cycling. Size-fractionated studies based on phytoplankton size classes [3] show that microphytoplankton (>20 μm) tend to dominate under nutrient-rich conditions and drive the classical food web and biological pump via large-particle formation and sinking or mesozooplankton grazing [4]. Nanophytoplankton (2–20 μm) and picophytoplankton (0.2–2 μm), on the other hand, are more tightly linked to the microbial loop [5]; most of the carbon they fix is respired within the euphotic zone, yielding low export efficiency [6]. Hence, total PP alone fails to reveal the full picture of carbon cycling—the spatiotemporal distribution of size-fractionated PP is essential for accurately assessing carbon-flow pathways.

**Figure 1:**
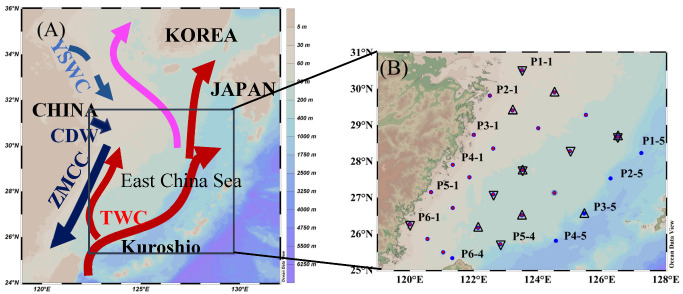
The area indicated by the blue rectangle in panel (**A**) shows the location of the study area in the East China Sea, and panel (**B**) shows the primary production sampling stations and bathymetry in the East China Sea. Chlorophyll-a sampling stations in December are indicated by red circles, and those in February are indicated by blue circles. PP stations sampled in December and February are marked by downward-pointing triangles and upward-pointing triangles, respectively, while stations sampled during both cruises are marked by diamonds.

The East China Sea (ECS) is a typical marginal sea of the northwestern Pacific, with a broad continental shelf (Figure 1A) [7]. Monsoonal forcing and a complex circulation system impart distinct winter hydrographic and dynamic features. The Changjiang Diluted Water (CDW), carrying cold, low-salinity coastal water, is transported southward by coastal currents and forms the narrow southward-flowing Zhe–Min Coastal Current (ZMCC) along the coast [8]. Over the central and eastern shelf, the Taiwan Warm Current (TWC) and its branches flow northeastward [9], while the Kuroshio runs northward along the continental slope at the outer shelf margin [10].

Enhanced winter monsoon forcing deepens vertical mixing, often pushing the mixed layer depth below the euphotic depth. This process transports nutrients upward but simultaneously reduces light availability in the water column [11]. As a result, winter PP is generally at its annual minimum [12,13]. Despite these low levels, both the magnitude and size structure of winter PP carry considerable ecological significance. The ECS shelf serves as an important overwintering ground for commercially valuable fish such as jack mackerel and mackerels [14,15]. Winter PP supplies the basal energy, while its size structure may indirectly regulate energy available to larval fish by shaping the food sources for mesozooplankton [4,16].

Current understanding of winter PP in the ECS, however, is limited in two key respects. First, field-based observational evidence on the mechanisms underlying low productivity remains sparse. Gong and Liu (2003) [17] and Liu et al. (2010) [11] suggested—via empirical and coupled models, respectively—that light availability is the primary limiting factor. Yet these conclusions are based on model simulations or observations confined to local areas, and direct cross-shelf field evidence linking low PP to light limitation is still lacking. Second, the size structure of winter PP is poorly known. Existing size-fractionated studies have focused mainly on spring and summer [18,19] or have relied on satellite remote-sensing retrievals [20,21]. Although Gong et al. (2017) [22] measured P–E curves in the ECS during winter and estimated euphotic-zone integrated PP, they did not partition contributions by size class. Consequently, the relative roles of different phytoplankton size classes in NPP and their implications for food-web energy transfer remain unclear.

To address these gaps, we conducted two winter field cruises in the ECS. We identified key factors regulating winter NPP and characterized the spatiotemporal variability of size-fractionated NPP and its contribution to total productivity. We also used field observations to examine variations in key parameters from several widely applied satellite-based NPP models and conducted a comparative evaluation of these models.

## 2. Materials and Methods

This study was based on two winter cruises conducted aboard the research vessel Beidou in the southern East China Sea (25–33° N, 120–127.5° E). The first cruise was conducted from 19 November to 23 December 2006, hereafter referred to as the December cruise, and the second from 22 February to 11 March 2007, hereafter referred to as the February cruise. Sampling stations were arranged along six transects. In total, 110 stations were sampled for chlorophyll-a and environmental parameters, with 55 stations during each cruise, and 14 stations were selected for primary production measurements, with 7 stations during each cruise (Figure 1B). The primary production stations were selected to adequately represent the salinity and water-depth gradients from the nearshore region to the outer shelf.

### 2.1. Environmental Parameters

At each station, temperature and salinity were measured using a CTD profiler (Sea-Bird Electronics) mounted on a rosette sampler equipped with 2.5 L Niskin bottles. Dissolved inorganic nitrogen (DIN, including NOx and NH_4_^+^), PO_4_-P, and SiO_3_-Si were measured using a Skalar San analyzer following the National Standard of the People’s Republic of China (GB12763.4-91) [23]. Nutrient limitation was assessed based on a combination of stoichiometric criteria (Justić et al., 1995) [24] and empirical concentration thresholds. Specifically, DIN < 1 μmol L^−1^, PO_4_-P < 0.1 μmol L^−1^, and SiO_3_-Si < 2 μmol L^−1^ were used as potential limitation thresholds for nitrogen, phosphorus, and silicon, respectively. These threshold values were derived from studies of nutrient uptake kinetics and have been widely applied in coastal systems [25].

Light exposure (LE) was calculated from surface irradiance, namely photosynthetically available radiation (PAR), obtained from the NASA Ocean Color database (https://oceancolor.gsfc.nasa.gov) at a spatial resolution of 4 km and daily temporal resolution, following Falkowski (1981) [26]:

$$LE=\frac{I_{0}}{MLD\cdot K}\times \left(1-e^{-MLD\cdot K}\right)$$

The optimal chlorophyll-specific carbon fixation rate, P^B^_opt_, was calculated from volumetric primary production and chlorophyll-a concentration at the same depth within the euphotic zone:

$$P^{B}\left(z_{i}\right)=\frac{{PP(z}_{i})}{Chl(z_{i})}$$

The water-column P^B^_opt_ was defined as the maximum value of P^B^(z) among the corresponding light depths within the euphotic zone, where zi ∈ [0, Z_eu_]:

$$P_{opt}^{B}=\underset{z\in \left[0,Z_{eu}\right]}{max}P^{B}\left(z\right)$$

The dimensionless factor F was derived from euphotic-zone integrated primary production, euphotic depth, and the optimal chlorophyll-specific carbon fixation rate, following Behrenfeld and Falkowski (1997) [27]:

$$F=\frac{{PP}_{int}}{{P_{opt}^{B}\times C}_{opt}\times Z_{eu}\times D_{irr}}$$

where (I_0_) is surface irradiance (Einstein m^−2^ d^−1^), (MLD) is the mixed layer depth (m), and (K) is the light attenuation coefficient. PP_int_) is the depth-integrated phytoplankton primary production within the euphotic zone (mg C m^−2^ d^−1^), and (Chl_int_) is the depth-integrated chlorophyll-a within the euphotic zone (mg Chl a m^−2^). (PP(z_i_)) represents volumetric primary production at depth (z_i_) (mg C m^−3^ h^−1^), and (Chl(z_i_)) is the chlorophyll-a concentration at the same depth (mg m^−3^). (C_opt_) is the chlorophyll-a concentration at the depth where P^B^_opt_ occurs (mg m^−3^); (Z_eu_) is the euphotic depth (m), estimated from Secchi disk depth(Z_sd_) using the empircial relationship Z_eu_ = Z_sd_ × 2.7 [28,29]; and (D_irr_) is day length (h d^−1^). The unit of (P^B^(z_i_)) is mg C (mg Chl a)^−1^ h^−1^. In this study, (MLD), which is related to water-column stability, was defined as the first depth at which temperature decreased by 0.5 °C relative to the surface value [30,31].The light attenuation coefficient (K) was calculated from Secchi disk depth using the method proposed by Castillo-Ramírez et al. (2020) [32].

### 2.2. Chlorophyll-a

Chlorophyll-a concentration (mg·m^−3^) was measured fluorometrically [33]. During the cruises, size-fractionated filtration was conducted to determine chlorophyll-a concentrations in different phytoplankton size classes. At each station and sampling depth, 200 mL of seawater was collected and first passed through a 200 μm nylon mesh to remove zooplankton. The sample was then filtered through a size-fractionation filtration system in which 20 μm filters for microphytoplankton (>20 μm) (MA, USA), 2 μm filters for nanophytoplankton (2–20 μm) (MA, USA), and Whatman GF/F filters (0.7 μm) (MA, USA) for picophytoplankton (<2 μm) were arranged sequentially from top to bottom [3].

Filters from all size fractions were extracted overnight in 90% acetone at −20 °C in the dark, and chlorophyll-a content was measured using the same Trilogy fluorometer. Chlorophyll-a concentrations for each size fraction were calculated by dividing the measured chlorophyll-a content by the corresponding filtered volume, and total chlorophyll-a concentration was obtained as the sum of the three size fractions.

### 2.3. Net Primary Production

Net primary production (NPP) was measured using the ^14^C isotope tracer method originally established by Nielsen (1952) [34] and subsequently modified by Ning et al. (1988) [35]. Seawater samples were collected from six light depths within the euphotic zone. Prior to incubation, the samples were gently passed through a 200 μm mesh to remove zooplankton and then dispensed into two light bottles and one dark bottle. A known amount of NaH^14^CO_3_ (Shanghai, China) tracer was added to each bottle.

The incubation system was maintained at a nearly constant temperature by continuously circulating surface seawater. Six relative irradiance levels, corresponding to 100%, 50%, 30%, 10%, 3%, and 1% of surface incident irradiance, were established using neutral-density screens of different transmittances. These light levels corresponded to different optical depths within the euphotic zone and were used to simulate the in situ light environment experienced by phytoplankton. The incubation bottles were placed on the ship deck and incubated under simulated in situ conditions close to the surface seawater temperature for 6 h.

After incubation, seawater from each bottle was sequentially filtered under low vacuum pressure (<0.02 MPa) through 20 μm filters, 2 μm filters, and Whatman GF/F filters (0.7 μm). The particulate matter retained on each filter represented the micro-, nano-, and pico-phytoplankton fractions, respectively. The filters were fumed with concentrated hydrochloric acid to remove inorganic ^14^C and then dried in scintillation vials and stored at low temperature. After the cruise, radioactivity was measured in the laboratory using a PE 2900 liquid scintillation counter. PP was calculated according to the equation recommended by Parsons et al. (1984) [36].

### 2.4. Data Analysis

Spatial distribution maps were generated using Ocean Data View (version 5.2.1) and Python (version 3.9). Prior to principal component analysis (PCA), all variables were standardized using z-score normalization to eliminate the influence of differences in units and scales. Differences in NPP between the December and February cruises were tested using the Mann–Whitney U test, and Kruskal–Wallis tests were used for multi-group comparisons among depth zones. Group separation in PCA space was tested using PERMANOVA (9999 permutations, Euclidean distance). Bootstrap resampling (*n* = 10,000) was used to estimate 95% confidence intervals for mean values in each depth zone. All statistical analyses were performed in Python (version 3.9).

## 3. Results

### 3.1. Hydrographic Conditions and Light Field in the Study Area

**Figure 2:**
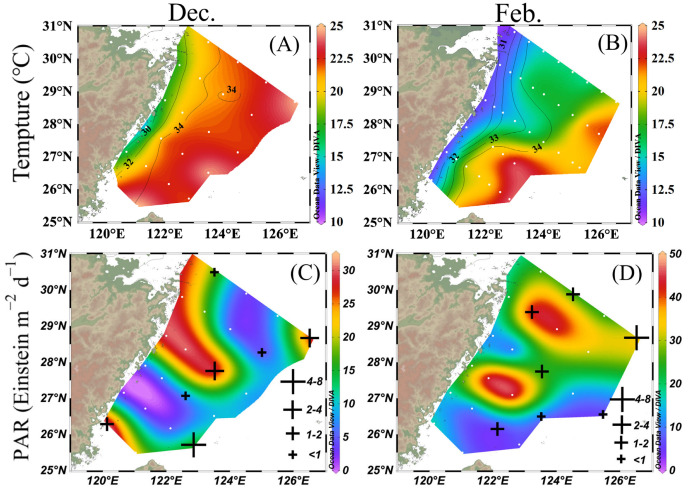
Spatial distributions of hydrographic and optical parameters in the East China Sea during winter. The left panels (**A**,**C**) and right panels (**B**,**D**) represent the cruises conducted in December 2006 and February 2007, respectively. (**A**,**B**) Sea surface temperature (colored shading) and salinity contours (black solid lines). (**C**,**D**) Surface photosynthetically available radiation (PAR; colored shading). Cross symbols (+) indicate mixed-layer mean light exposure (LE, Einstein m^−2^ d^−1^), with symbol size proportional to LE intensity; white circles indicate stations with no available data.

Surface temperature and salinity during the two winter cruises displayed clear cross-shelf gradients, with lower values nearshore and higher values offshore (Figure 2A,B; Table 1). Mean surface temperature was 21.15 ± 2.59 °C in December and dropped to 18.12 ± 4.11 °C in February, while mean salinity rose slightly from 33.14 ± 1.59 to 33.46 ± 1.89 psu. Low-temperature water expanded toward the inner shelf and the northern part of the study area. Salinity distributions showed that low-salinity water remained largely confined nearshore, whereas the middle and outer shelves were occupied by high-salinity water.

**Figure 3:**
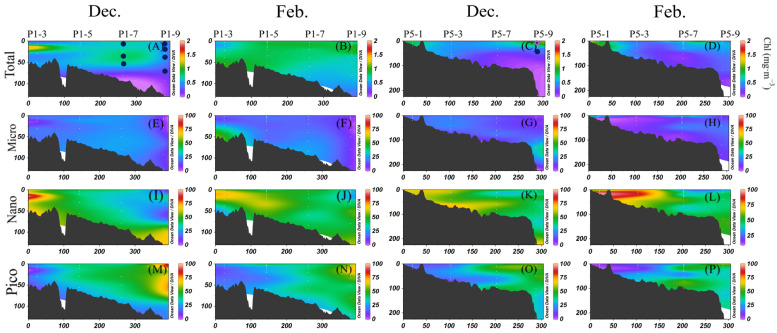
Vertical distributions of chlorophyll-a concentration and the relative contributions of different size fractions along two representative transects (P1 and P5) in the East China Sea during winter. (**A**–**D**) Total chlorophyll-a concentration (mg m^−3^); (**E**–**H**) relative contribution of the micro-sized fraction; (**I**–**L**) relative contribution of the nano-sized fraction; (**M**–**P**) relative contribution of the pico-sized fraction to total chlorophyll-a (%).

Nutrient concentrations at each station are presented in Supplementary Table S1. DIN, PO_4_-P, and SiO_3_-Si concentrations generally exceeded potential limitation thresholds at the majority of stations, consistent with the nutrient profiles shown in Figure 3. The calculated N:P ratios at most stations exceeded the Redfield ratio of 16 (Appendix A Table A1), further confirming that nutrients were not a widespread limiting factor for phytoplankton growth during winter.

The underwater light field, by contrast, exhibited pronounced cross-shelf heterogeneity (Figure 2C,D). Euphotic depth increased markedly from 11.29 ± 4.41 m on the inner shelf to 28.73 ± 1.16 m on the outer shelf (Table 1), reflecting a gradual offshore decline in the light attenuation coefficient. PAR displayed the reverse pattern: Values were relatively high over the inner shelf (29.46 ± 10.53 Einstein m^−2^ d^−1^) and lowest over the outer shelf (10.57 ± 6.46 Einstein m^−2^ d^−1^).

### 3.2. Vertical Distribution of Chl and Size Structure of the Phytoplankton Community

Along transect P1, chlorophyll-a sections showed that in December, Chl concentrations were highest in the upper water column, with a pronounced maximum of 1.44 mg m^−3^ in the nearshore upper layer of the inner shelf. Concentrations decreased offshore and with depth, and low values prevailed over the outer shelf and in deeper waters. In February, Chl-a concentrations in the nearshore upper layer along transect P1 were notably lower than in December, while relatively elevated values were observed in the mid-shelf upper water column. The overall cross-shelf gradient was less pronounced compared to the December cruise.

The nano-sized fraction dominated nearshore upper-layer Chl. Moving offshore, the pico-sized fraction gradually increased and became dominant in the upper layer of the outer shelf, whereas the micro-sized fraction contributed relatively little to total Chl.

Along transect P5, Chl concentrations were overall lower than along P1. The size structure was again dominated by the nano-sized fraction, followed by the pico-sized fraction, and the micro-sized fraction contributed least.

### 3.3. Size-Fractionated Primary Production Structure and Contributions

**Figure 4:**
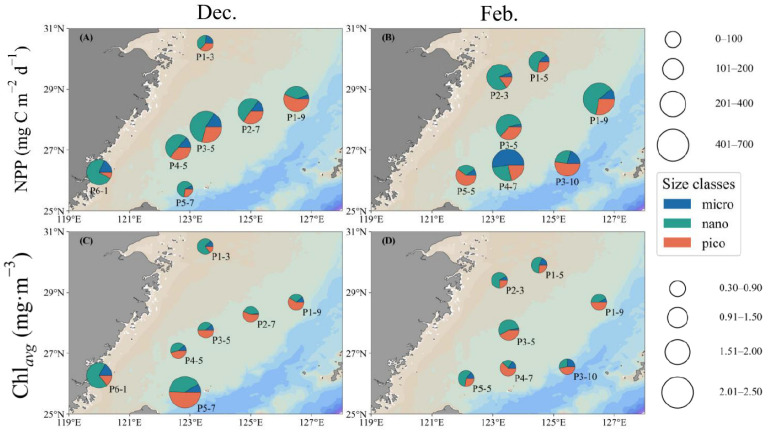
Spatial distributions of size-fractionated primary production and chlorophyll-a in the East China Sea during winter. (**A**,**B**) Depth-integrated primary production (NPP, mg C m^−2^ d^−1^); (**C**,**D**) water-column mean chlorophyll-a concentration (Chl *a*, mg m^−3^). The left panels (**A**,**C**) correspond to the December cruise, and the right panels (**B**,**D**) correspond to the February cruise.

NPP was higher at inner- and middle-shelf stations and generally lower over the outer shelf (Table 1). In December, depth-integrated total primary production ranged from 16.09 to 525.56 mg C m^−2^ d^−1^ (Figure 4A), with nanophytoplankton contributing ~54%, picophytoplankton 32%, and microphytoplankton 14%.

February NPP ranged from 114.02 to 626.08 mg C m^−2^ d^−1^ (Figure 4B) and was generally higher than in December. Nanophytoplankton remained dominant at most stations, contributing 48% of total NPP and reaching 60–80% or more over the northern and middle shelf. The relative contribution of picophytoplankton increased to 29% over the southern shelf slope and at several offshore stations. Microphytoplankton contributed 23%, up from 14% in December. A Mann–Whitney U test showed no significant difference in NPP between the two cruises.

Depth-integrated Chl decreased slightly from nearshore to offshore in December. The nano-sized fraction dominated total Chl, the pico-sized fraction contributed relatively more at outer-shelf stations, and microphytoplankton made only a minor overall contribution. In February, Chl increased relative to December, particularly at middle-shelf stations, with nano-sized algae remaining dominant but pico-sized contributions rising markedly at some stations (Figure 4D).

### 3.4. Principal Component Analysis of Environmental Factors, Chl, and PP

**Figure 5:**
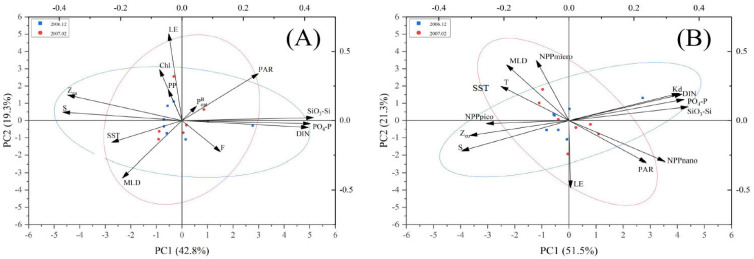
Principal component analysis (PCA) of environmental factors and biological parameters in the East China Sea during winter. (**A**) PCA of environmental factors, chlorophyll-a (Chl), and total primary production; (**B**) PCA of environmental factors and the relative contributions of size-fractionated primary production.

Principal component analysis (PCA) showed that the first two principal components explained 42.8% and 19.3% of the total variance, respectively (Figure 5A). PC1 captured the cross-shelf physicochemical gradient: DIN, PO_4_-P, and SiO_3_-Si loaded positively on PC1, while salinity and euphotic depth loaded negatively, reflecting the transition from nearshore waters with high nutrient concentrations and high turbidity to saltier, clearer outer-shelf waters. PC2 represented the coupling between the light field and mixing processes—NPP and Chl covaried positively with LE and negatively with MLD, highlighting light availability as a key factor explaining winter NPP variability in the East China Sea.

In the size-fractionated PCA (Figure 5B), the contribution of picophytoplankton to NPP tracked salinity and euphotic depth, signaling a higher relative contribution in the high-salinity, deep-euphotic-zone waters of the outer shelf. Nanophytoplankton contribution aligned with the positive direction of PC1, consistent with nearshore, high-nutrient, high-turbidity conditions. The contribution of microphytoplankton to NPP was positively associated with SST along PC2.

## 4. Discussion

### 4.1. Winter NPP and Environmental Controls

Across the two cruises, mean NPP was 247.12 ± 156.99 mg C m^−2^ d^−1^ in December and 316.35 ± 171.01 mg C m^−2^ d^−1^ in February. These values sit at the low end of the annual range, representing less than one-third of the summer shelf value reported for the East China Sea (939 mg C m^−2^ d^−1^ [13]), and align with earlier reports of low winter productivity in the region [12,22].

Previous studies have offered several explanations for low winter productivity. Gong and Liu (2003)) [17] argued that light is the primary limiting factor for phytoplankton growth in the East China Sea during winter. Using a coupled physical–biogeochemical model, Liu et al. (2010) [11] further suggested that winter deepening of the mixed layer beyond the euphotic depth reduces the mean light experienced by phytoplankton cells, thereby suppressing primary production. Zhu et al. (2024) [37], working with simultaneous fast repetition rate fluorescence (FRRF) parameters and carbon uptake rates in the Changjiang Estuary and adjacent East China Sea during summer, found that NPP was closely associated with Chl a and PSII-related parameters, suggesting that in a dynamically complex shelf sea such as the ECS, the variability of phytoplankton productivity is linked to both phytoplankton standing stock and photosynthetic physiological status, while the primary regulatory factors remain light penetration depth, nutrient availability, temperature, and day length.

By combining concurrent observations of nutrients, light conditions, and P^B^_opt_, we present three lines of evidence that light is the primary limiting factor for winter primary production in the East China Sea. First, nutrient concentrations at most stations exceeded potential limitation thresholds, with only a few nearshore stations showing potential N, P, or Si limitation (Figure 3), indicating that nutrient availability was not the dominant constraint. Second, LE at most stations fell below the diatom light-saturation threshold of ~4 Einstein m^−2^ d^−1^, suggesting that ambient light conditions were insufficient for photosynthesis to reach its maximum rate and thus were unfavorable for bloom development [38]. PCA results showed that NPP varied positively with LE and negatively with MLD (Figure 5A), highlighting that water-column light availability explained much of the spatial variability in NPP. More importantly, observed P^B^_opt_ averaged 2.28 ± 1.34 mg C (mg Chl a)^−1^ h^−1^ in December and 2.16 ± 1.23 mg C (mg Chl a)^−1^ h^−1^ in February, both lower than the values predicted by the VGPM model based on contemporaneous SST (3.11 ± 2.28 mg C (mg Chl a)^−1^ h^−1^). This overestimation indicates that SST-based P^B^_opt_ prediction fails to capture the suppression of photosynthetic rates under the low-light conditions caused by deep winter mixing. Collectively, these three lines of evidence demonstrate that deep vertical mixing, by limiting light availability in the water column, largely drove the low winter productivity.

Although mean NPP was numerically higher in February than in December, the difference between the two cruises was not statistically significant; thus, we cannot confirm a systematic recovery in productivity from December to February. Nevertheless, LE at several outer-shelf stations in February approached the light-saturation threshold (Figure 2D), and nearshore upper-layer Chl along transect P1 was higher than in December (Figure 3), consistent with the seasonal increase in PAR. Xu et al. (2022) [39] reported that the spring increase in chlorophyll in the East China Sea is typically associated with sea surface warming, increased PAR, and enhanced stratification from weakened wind-driven mixing. Gong et al. (1997) [40] also found that off northeastern Taiwan, chlorophyll rose rapidly after spring upwelling resumed following the weakening of Kuroshio intrusion. These patterns suggest that light limitation may have partially eased in some areas by late winter, positioning February as a transitional stage between the low-productivity winter period and the spring phytoplankton growth season.

### 4.2. Size Structure of Winter NPP in the East China Sea and Its Ecological Implications

Knowledge of phytoplankton size structure in the East China Sea comes largely from spring and summer field observations or satellite remote-sensing retrievals. Chen (2000) [18] compared shelf water, upwelling water, and Kuroshio water in the southern East China Sea and found that PP was lowest in Kuroshio water, where the pico-sized fraction dominated, and highest in the upwelling region, where the micro-sized fraction contributed most. Using pigment data, Furuya et al. (2003) [19] reported that diatoms dominated the East China Sea shelf in spring and summer. Satellite-based work by Sun et al. (2018) [20] and Zhang et al. (2018) [21] further showed that coastal waters generally had a higher micro-sized contribution year-round, the Kuroshio region was mostly dominated by the pico-sized fraction, and the middle shelf was dominated by the micro- and nano-sized fractions during winter and spring. However, in situ size-fractionated ^14^C-based PP measurements in the East China Sea during winter remain scarce.

We provide field observations of size-fractionated NPP in the East China Sea in winter. The nano-sized fraction dominated NPP in both cruises, accounting for 53.77% in December and 48.67% in February, followed by the pico-sized fraction (32.26% and 29.55%, respectively). The micro-sized fraction contributed least—13.96% in December and 21.78% in February (Figure 4). This pattern differs from the micro-dominated structure commonly observed over the shelf in spring and summer and from the pico-dominated structure typical of the Kuroshio region, reflecting a distinct phytoplankton community structure under winter light-limited conditions. The offshore dominance of the pico-sized fraction may also reflect the influence of Kuroshio water intrusion, which transports oligotrophic, picophytoplankton-dominated communities onto the outer shelf of the ECS [41].

**Figure A1:**
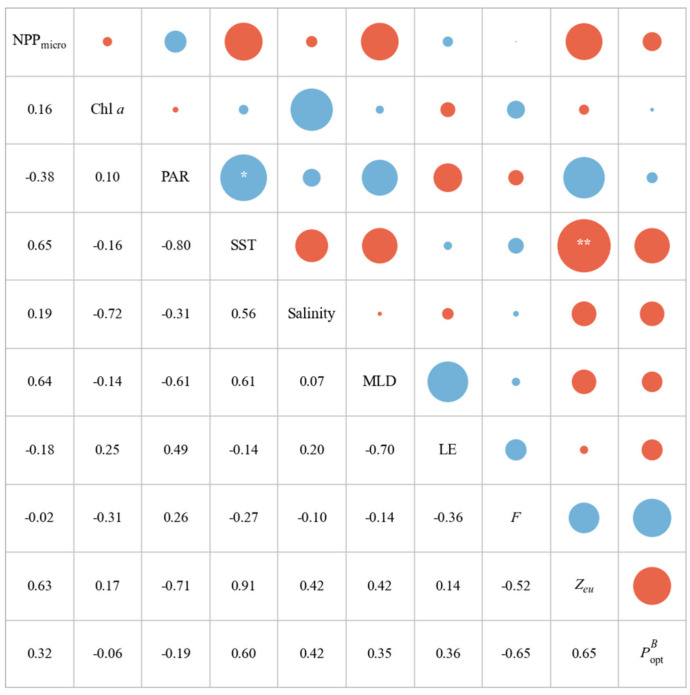
Pearson correlation matrix between micro-sized net primary production and environmental variables in the East China Sea in February (* *p* < 0.05; ** *p* < 0.01).

**Figure A2:**
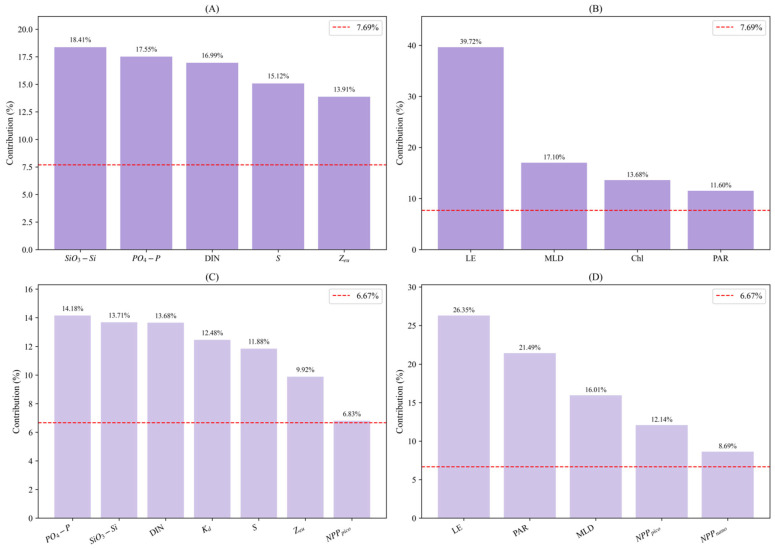
Percentage contributions of variables to the first two principal components (PC1 and PC2) of the two PCA analyses. (**A**,**B**) PCA of environmental variables (corresponding to Figure 5A); (**C**,**D**) PCA of environmental and biological variables (corresponding to Figure 5B). Only variables with contributions exceeding the average expected contribution (red dashed line: 7.69% for 13 variables in Group 1; 6.67% for 15 variables in Group 2) are considered important contributors to each component.

The micro-sized contribution to NPP showed a modest increase from 13.96% in December to 21.78% in February, though this difference was not statistically significant (Mann–Whitney U test, *p* > 0.05). PCA results suggested that this contribution varied in the same direction as SST and MLD (Figure 5B), indicating a possible, albeit not statistically confirmed, trend toward a greater relative role for larger phytoplankton in late winter. Correlation analysis further indicated that the micro-sized contribution to NPP was positively correlated with SST, MLD, and Z_eu_ (Appendix A Figure A1 and Figure A2). From December to February, although SST declined, MLD deepened and euphotic depth increased at some stations. These changes may have strengthened the relative contribution of microphytoplankton: Deeper late-winter mixing can transport more nutrients from subsurface layers into the euphotic zone, and larger phytoplankton generally have a competitive edge under nutrient-replete conditions and can respond more rapidly when light improves [42,43]. In addition, LE at some February stations approached the light-saturation threshold; this partial relief of light limitation may have preferentially favored microphytoplankton, whose photosynthetic rates are typically more sensitive to changes in light availability than those of pico- and nanophytoplankton [44].

Shifts in phytoplankton size structure carry potential implications for food-web energy transfer [4,6]. Larger phytoplankton are more likely to enter the classical food web through grazing by mesozooplankton such as copepods [45,46], whereas pico- and nanophytoplankton are more closely tied to microbial food-web cycling [5,6]. The East China Sea shelf serves as an important overwintering and spawning ground for commercially valuable fish such as jack mackerel and mackerels [14,15], and their larvae prey mainly on small zooplankton and copepods in the 50–100 μm size range [47]. The modest increase in micro-sized NPP observed in February coincided temporally with the late overwintering period and the pre-spawning stage of these species [14]. Although the potential link between late-winter increases in micro-sized production and prey availability for fish larvae is ecologically plausible, this hypothesis remains speculative in the absence of concurrent zooplankton and ichthyoplankton observations and requires verification through future integrated studies.

A further pattern worth noting is the decoupling between Chl contribution and NPP contribution across size fractions (Figure 4). In February, for instance, the pico-sized fraction accounted for 47.08% of total Chl but only 29.55% of total NPP, whereas the micro-sized fraction accounted for 13.83% of total Chl but contributed 21.78% of total NPP, pointing to a biomass–productivity decoupling among size classes. The growth–loss framework proposed by Westberry et al. (2016) [48] offers a possible explanation: A given size fraction can maintain relatively high productivity despite a low Chl standing stock if it has high photosynthetic activity and a rapid turnover rate [49]. Conversely, a fraction with a large Chl stock may not contribute proportionally to NPP because loss processes such as grazing and sinking are unevenly distributed across size classes [50]. Studies have shown that microzooplankton can consume roughly 49–77% of daily marine primary production [51] and that grazing is strongly size-selective [52]. Photoacclimation to the low-light winter environment may also contribute to the observed biomass–productivity decoupling, as phytoplankton under sub-saturating light conditions tend to increase their cellular Chl-a content [53], elevating Chl-a relative to carbon fixation rates. Estimating winter primary production and its size-fractionated contributions in the East China Sea solely from satellite-derived Chl data may therefore introduce biases, and future work should incorporate in situ size-fractionated NPP measurements for validation.

### 4.3. Regional Applicability and Limitations of P^B^_opt_ Parameterization Models on the East China Sea Shelf

P^B^_opt_ is a key physiological parameter in satellite-based primary production models, and its parameterization directly affects water-column PP estimates [27,54]. In temperate waters, P^B^_opt_ generally falls within 1–6 mg C (mg Chl a)^−1^ h^−1^, but it is not a fixed constant—temperature, chlorophyll concentration, phytoplankton community structure, and other factors jointly regulate it [27,55,56,57,58]. Parameterization of P^B^_opt_ therefore critically shapes the accuracy of regional satellite-based PP estimates.

Current mainstream models predict P^B^_opt_ mainly through empirical relationships. The VGPM [27] expresses P^B^_opt_ as a function of SST. Kameda and Ishizaka (2005) [55] proposed a two-phytoplankton-community model that uses SST and surface Chl as inputs, and Gong and Liu (2003) [17] developed a regional empirical model for the East China Sea that incorporates the light attenuation coefficient and irradiance.

**Figure 6:**
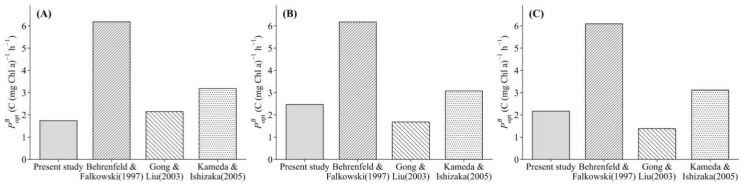
Comparison between model-predicted and observed P^B^_opt_ values in different subregions of the East China Sea shelf: (**A**) <50 m; (**B**) 50–200 m; and (**C**) >200 m [17,27,55].

We compared model-predicted P^B^_opt_ with our observations and found marked differences in performance among shelf subregions. The VGPM systematically overestimated P^B^_opt_ across all three depth zones (<50 m, 50–200 m, and >200 m; Figure 6), indicating a consistent positive bias for the winter East China Sea. The Gong and Liu (2003) [17] model showed relatively small bias on the inner shelf (<50 m) but greater scatter on the middle shelf (50–200 m). The Kameda and Ishizaka (2005) [55] model agreed more closely with observations on the middle shelf yet exhibited larger deviations on the inner and outer shelves. These results suggest that the controls on P^B^_opt_ differ among shelf subregions during winter, and no single parameterization scheme performs equally well across the entire shelf. Station-level comparison (*n* = 14) showed that none of the three models adequately predicted the observed P^B^_opt_ variability, with negative R^2^ values for all models, indicating that the models explained less variance than the observed mean. VGPM showed the largest overestimation (bias = +3.95, RMSE = 4.17), while Gong (2003) [17] and Yamada (2005) [56] showed smaller but still substantial errors (RMSE ≈ 1.5).

The VGPM’s systematic overestimation likely stems from the East China Sea’s distinctive winter light environment. The model assumes that temperature primarily governs P^B^_opt_, but deep winter mixing forces the mixed layer well below the euphotic depth, curtailing mean light exposure for phytoplankton. As a result, actual photosynthetic rates fall below predictions based solely on SST [58]. The Gong model’s relatively good performance on the inner shelf may reflect its inclusion of the light attenuation coefficient, which partly captures the influence of water-column optical conditions on P^B^_opt_. However, increased scatter on the middle and outer shelves indicates that the empirical relationship has limited general applicability across differing optical environments. Overall, existing P^B^_opt_ schemes based primarily on SST or SST–Chl relationships show clear limitations for the winter East China Sea. Future regional models should incorporate water-column light conditions to improve satellite-based PP estimates during winter. In addition, the VGPM model was calibrated using a global dataset that may not adequately represent the phytoplankton community composition of the ECS in winter, where nano-sized phytoplankton dominate rather than the diatom-dominated communities typical of many temperate regions. Community-specific photosynthetic parameters could differ from the global mean SST–P^B^_opt_ relationship, partly explaining the observed discrepancy.

### 4.4. Spatial Variation in F

Within the VGPM framework, the F value normalizes the surface maximum photosynthetic rate to the entire euphotic zone. F reflects the degree of vertical uniformity in photosynthetic rates within the euphotic zone: The closer F is to 1, the smaller the difference in photosynthetic rates among depths; the lower the F value, the more strongly photosynthesis is concentrated near the surface [54]. Vertical mixing, the relationship between euphotic depth and mixed layer depth, and phytoplankton photoacclimation characteristics jointly influence F [59,60,61]. Under strong mixing or shallow-water conditions, photosynthetic rates tend to vary less with depth, and F approaches 1. Conversely, in stratified waters with a deeper euphotic zone, photosynthesis remains largely confined to the upper layer, yielding lower F values [62,63].

We observed Ftotal values of 0.91 ± 0.34, 0.71 ± 0.29, and 0.38 ± 0.05 in the <50 m, 50–200 m, and >200 m depth zones, respectively (Table 1), with values declining from nearshore to offshore. On the shallow inner shelf, F approached 1, consistent with water depths where the euphotic depth nearly equaled total depth and virtually the entire water column lay within the effective light field. Over the outer shelf, F dropped to about 0.4, indicating that photosynthesis was largely concentrated in the upper euphotic zone. These patterns match winter observations from temperate shelves [51,54].

PCA results showed no positive covariation between F and MLD (Figure 5). Two factors may explain this. First, many stations lay on the nearshore shallow shelf, where the water column was often fully mixed and MLD approached total water depth, making interstation differences in MLD small and the influence of mixing intensity on F difficult to detect. Second, the shelf-to-slope region of the East China Sea is strongly shaped by interactions between the Kuroshio and bottom topography; cross-shelf exchange, shelf-break upwelling, and local wind-driven mixing can all modify the vertical distribution of light [7,9,11], so the spatial variability of F reflects multiple superimposed processes. In this topographically and hydrodynamically complex shelf, future parameterizations of F should incorporate the ratio of Z_eu_ to MLD and vertical mixing intensity rather than relying solely on MLD as a single predictor.

## 5. Conclusions

Winter observations of primary production on the East China Sea shelf allowed us to characterize the size-fractionated structure of NPP, identify its main environmental controls, and evaluate representative satellite-based PP models for this region. The principal conclusions are:

(1) Low winter NPP in the East China Sea is driven primarily by light limitation. Intense winter mixing reduces light exposure in the water column; LE at most stations fell below the phytoplankton light-saturation threshold. Nutrients, by contrast, were generally non-limiting across the study area, confirming that insufficient light suppressed phytoplankton growth.
(2) The size structure of winter PP shifted seasonally. The micro-sized fraction increased in February, which may reflect enhanced nutrient supply from deeper late-winter mixing and locally improved light conditions, although this change was not statistically significant. This potential greater contribution from larger phytoplankton could indicate a relative strengthening of the classical food-chain pathway, which may improve the prey field for fish during spring feeding.
(3) The widely applied VGPM model overestimated both key parameters—P^B^_opt_ and F—relative to our field measurements. The discrepancies varied with water depth, being smaller on the shallow inner shelf and progressively larger over the middle and outer shelves, a pattern linked mainly to regional differences in the mixed-layer-depth to euphotic-depth ratio.

A single SST-based parameterization cannot adequately represent the complex light environment of marginal sea shelves. Future work should integrate field observations from different subregions and include regionalized light-related parameters in the treatment of P^B^_opt_ and F, thereby strengthening satellite-based PP estimates for winter marginal seas.

## Figures and Tables

**Table 1:** Mean, standard deviation (SD), and bootstrap 95% confidence intervals (CI; *n* = 10,000 iterations) of size-fractionated primary production parameters across three depth zones in the East China Sea during winter.

Parameters	<50 m (*n* = 4)		50–200 m (*n* = 8)		>200 m (*n* = 2)	
	Mean ± SD	95% CI	Mean ± SD	95% CI	Mean ± SD	95% CI
NPPmicro	25.36 ± 21.63	[10.99, 46.57]	72.18 ± 107.07	[23.31, 151.14]	22.44 ± 27.72	[2.84, 42.04]
NPPnano	144.40 ± 116.87	[46.70, 242.09]	168.49 ± 85.66	[111.95, 224.90]	41.28 ± 19.70	[27.35, 55.21]
NPPpico	31.62 ± 17.05	[18.36, 45.60]	121.16 ± 34.63	[98.47, 143.68]	58.35 ± 66.74	[11.16, 105.55]
NPPtotal	201.37 ± 142.89	[82.99, 319.76]	361.83 ± 165.44	[258.01, 470.87]	122.07 ± 114.16	[41.35, 202.80]
Chl_avg_micro	0.14 ± 0.10	[0.08, 0.21]	0.05 ± 0.02	[0.05, 0.07]	0.15 ± 0.09	[0.07, 0.08]
Chl_avg_ nano	0.64 ± 0.31	[0.48, 0.92]	0.29 ± 0.16	[0.16, 0.33]	0.55 ± 0.63	[0.10, 0.38]
Chl_avg_ pico	0.18 ± 0.06	[0.13, 0.21]	0.31 ± 0.13	[0.19, 0.32]	0.69 ± 0.75	[0.16, 0.52]
Chl_avg_total	0.97 ± 0.44	[0.22, 0.77]	0.65 ± 0.26	[0.47, 0.71]	1.38 ± 1.47	[0.33, 0.97]
P^B^_opt_ micro	2.00 ± 1.13	[0.69, 1.56]	3.23 ± 2.24	[1.91, 4.73]	1.93 ± 2.19	[0.38, 3.48]
P^B^_opt_ nano	1.91 ± 1.34	[0.17, 1.71]	3.88 ± 2.00	[2.62, 5.21]	2.14 ± 2.42	[0.43, 3.85]
P^B^_opt_ pico	1.52 ± 0.55	[1.12, 2.00]	1.78 ± 0.63	[1.36, 2.18]	2.29 ± 2.92	[0.23, 4.36]
P^B^_opt_ total	1.74 ± 1.23	[0.89, 2.88]	2.47 ± 1.23	[1.64, 3.26]	2.17 ± 2.61	[0.33, 4.01]
C_opt_ micro	0.14 ± 0.12	[0.07, 0.26]	0.09 ± 0.07	[0.05, 0.14]	0.09 ± 0.00	[0.09, 0.09]
C_opt_ nano	0.68 ± 0.49	[0.38, 1.17]	0.30 ± 0.24	[0.18, 0.47]	0.30 ± 0.26	[0.11, 0.48]
C_opt_ pico	0.27 ± 0.12	[0.16, 0.36]	0.38 ± 0.27	[0.23, 0.57]	0.36 ± 0.24	[0.19, 0.54]
C_opt_ total	1.10 ± 0.64	[0.61, 1.73]	0.82 ± 0.47	[0.57, 1.16]	0.75 ± 0.51	[0.39, 1.11]
Fmicro	0.82 ± 0.33	[0.60, 1.15]	0.72 ± 0.39	[0.50, 0.99]	0.34 ± 0.13	[0.25, 0.43]
Fnano	0.90 ± 0.41	[0.66, 1.30]	0.59 ± 0.22	[0.46, 0.74]	0.41 ± 0.01	[0.40, 0.41]
Fpico	0.70 ± 0.26	[0.50, 0.96]	0.81 ± 0.45	[0.57, 1.14]	0.34 ± 0.09	[0.28, 0.40]
Ftotal	0.91 ± 0.34	[0.69, 1.22]	0.71 ± 0.29	[0.54, 0.92]	0.38 ± 0.05	[0.34, 0.41]
Z_eu_	11.29 ± 4.41	[7.39, 14.98]	28.32 ± 5.52	[24.73, 31.91]	28.73 ± 1.16	[27.91, 29.55]
SST	18.32 ± 3.27	[15.49, 21.15]	21.50 ± 2.90	[19.50, 23.22]	23.04 ± 0.99	[22.33, 23.74]
PAR	29.46 ± 10.53	[21.23, 38.68]	18.65 ± 11.06	[11.47, 25.72]	10.57 ± 6.46	[6.00, 15.14]

Note: 95% CI was derived using the percentile bootstrap method (10,000 resamples). For the >200 m zone (*n* = 2), bootstrap CI converges to the range of the two observations and should be interpreted with caution.

## Data Availability

The datasets generated and analyzed during the current study are available from the corresponding author upon reasonable request.

## Supplementary Materials

The following supporting information can be downloaded at: https://www.mdpi.com/article/10.3390/biology15120905/s1, Table S1: Nutrient concentrations at each station.

## Abbreviations

The following abbreviations are used in this manuscript:

NPP	Net primary production (mg C m^−2^ d^−1^)
PP	Primary production
Chl	Chlorophyll-a concentration (mg m^−3^)
P^B^_opt_	Optimal chlorophyll-specific carbon fixation rate (mg C (mg Chl a)^−1^ h^−1^)
C_opt_	Chlorophyll-a concentration at the depth of P^B_opt (mg m^−3^)
F	Dimensionless light-related factor for vertical distribution of primary production
Z_eu_	Euphotic depth (m)
MLD	Mixed layer depth (m)
LE	Mixed-layer mean light exposure (Einstein m^−2^ d^−1^)
PAR	Photosynthetically available radiation (Einstein m^−2^ d^−1^)
SST	Sea surface temperature (°C)
K	Light attenuation coefficient (m^−1^)
DIN	Dissolved inorganic nitrogen (μmol L^−1^)
ECS	East China Sea
CDW	Changjiang Diluted Water
ZMCC	Zhe–Min Coastal Current
TWC	Taiwan Warm Current
VGPM	Vertically generalized production model
PCA	Principal component analysis
FRRF	Fast repetition rate fluorescence

## Appendix A

**Table A1:** Nutrient stoichiometric ratios (N:P, N:Si, Si:P) at all stations during the December and February cruises in the East China Sea.

	Dec.			Feb.		
	N:P	N:Si	Si:P	N:P	N:Si	Si:P
P1−1	28.68	1.38	20.80	19.60	1.04	18.83
P1−2	28.99	1.25	23.14	20.49	1.09	18.76
P1−3	24.75	1.43	17.28	21.56	1.26	17.14
P1−4	48.07	2.69	17.88	18.84	0.85	22.06
P1−5	-	-	-	32.27	1.34	24.17
P2−1	29.24	1.01	29.05	43.05	1.47	29.33
P2−2	25.36	1.35	18.76	24.61	1.13	21.81
P2−3	30.29	1.79	16.93	22.56	1.12	20.22
P2−4	29.22	1.75	16.73	22.42	1.17	19.08
P2−5	-	-	-	30.71	1.59	19.33
P3−1	29.63	1.03	28.74	43.06	1.32	32.66
P3−2	28.76	1.48	19.45	34.03	1.07	31.75
P3−3	29.65	1.96	15.12	29.27	1.18	24.88
P3−4	34.50	2.31	14.96	21.67	1.16	18.67
P3−5	-	-	-	20.37	1.16	17.59
P4−1	22.53	0.83	27.06	27.24	0.87	31.42
P4−2	25.14	1.34	18.72	28.35	2.16	13.14
P4−3	31.17	1.73	17.97	33.17	1.69	19.59
P4−4	51.64	1.71	30.28	24.04	2.21	10.90
P4−5	-	-	-	31.42	1.19	26.36
P5−1	28.05	0.97	28.81	33.48	2.99	11.19
P5−2	30.98	1.51	20.50	22.02	1.57	14.05
P5−3	29.16	1.31	22.20	31.94	2.86	11.16
P5−4	22.76	1.32	17.28	37.95	3.38	11.21
P6−1	31.08	1.51	20.55	30.32	0.90	33.54
P6−2	36.67	1.71	21.50	31.57	1.36	23.25
P6−3	32.57	1.59	20.53	28.85	2.02	14.25
P6−4	-	-	-	28.71	1.63	17.66

Note: N:P, N:Si, and Si:P ratios were calculated from depth-integrated nutrient concentrations over the full water column at each station.
